# Concordance of emergency department physicians’ decisions on HIV post-exposure prophylaxis with national guidelines: results from a retrospective cohort study

**DOI:** 10.1093/inthealth/ihad076

**Published:** 2023-08-25

**Authors:** Johannes Heck, Christoph Höner zu Siederdissen, Olaf Krause, Sebastian Schröder, Martin Schulze Westhoff, Patrick-Pascal Strunz, Carsten Schumacher, Dirk O Stichtenoth, Jacobus J Bosch, Thorben Pape, Felix Koop, Benjamin Krichevsky

**Affiliations:** Hannover Medical School, Institute for Clinical Pharmacology, Carl-Neuberg-Str. 1, 30625 Hannover, Germany; Hannover Medical School, Emergency Department, Carl-Neuberg-Str. 1, 30625 Hannover, Germany; Hannover Medical School, Institute for General Practice and Palliative Care, Carl-Neuberg-Str. 1, 30625 Hannover, Germany; DIAKOVERE Henriettenstift, Center for Medicine of the Elderly, Schwemannstr. 19, 30559 Hannover, Germany; Hannover Medical School, Department of Psychiatry, Social Psychiatry and Psychotherapy, Carl-Neuberg-Str. 1, 30625 Hannover, Germany; Hannover Medical School, Department of Psychiatry, Social Psychiatry and Psychotherapy, Carl-Neuberg-Str. 1, 30625 Hannover, Germany; University Hospital Würzburg, Department of Internal Medicine II, Rheumatology and Clinical Immunology, Oberdürrbacher Str. 6, 97080 Würzburg, Germany; Hannover Medical School, Center for Clinical Trials, Feodor-Lynen-Str. 15, 30625 Hannover, Germany; Hannover Medical School, Institute for Clinical Pharmacology, Carl-Neuberg-Str. 1, 30625 Hannover, Germany; Centre for Human Drug Research, Zernikedreef 8, 2333 CL Leiden, The Netherlands; Leiden University Medical Center, Albinusdreef 2, 2333 ZA Leiden, The Netherlands; Hannover Medical School, Institute for Clinical Pharmacology, Carl-Neuberg-Str. 1, 30625 Hannover, Germany; Hannover Medical School, Institute for Clinical Pharmacology, Carl-Neuberg-Str. 1, 30625 Hannover, Germany; Technical University of Munich, Department of Internal Medicine II, Division of Clinical Toxicology, Ismaninger Str. 22, 81675 Munich, Germany; Hannover Medical School, Institute for General Practice and Palliative Care, Carl-Neuberg-Str. 1, 30625 Hannover, Germany

**Keywords:** drug safety, drug–drug interactions, emergency department, human immunodeficiency virus, internal medicine, post-exposure prophylaxis

## Abstract

**Background:**

Post-exposure prophylaxis (PEP) is an effective tool to prevent infection with HIV. Patients seeking PEP after potential HIV exposure usually present to the emergency department (ED). Our study sought to determine the concordance of ED physicians’ decisions on HIV-PEP with national guidelines (primary objective) and to assess the clinical relevance of drug–drug interactions (DDIs) between the HIV-PEP regimen and patients’ concomitant medication (secondary objective).

**Methods:**

We conducted a retrospective cohort study at the ED of Hannover Medical School, Germany. Between 1 January 2018 and 31 December 2019, 113 of 11 246 screened patients presented to the ED after potential HIV exposure and were enrolled in the study.

**Results:**

The median age of the patients (82.3% male) was 30 y (IQR 25–35.5), 85.8% of potential HIV exposures were characterised as sexual and 85.0% presented within 72 h. ED physicians’ decisions on HIV-PEP were concordant with national guidelines in 93.8%. No clinically relevant DDIs were detected.

**Conclusions:**

ED physicians’ decisions on HIV-PEP were highly concordant with national guidelines. Approximately 1% of patient presentations to the ED were related to HIV exposure; therefore, training ED physicians on HIV transmission risk assessment and indications/contraindications for HIV-PEP is paramount.

## Introduction

Application of antiretroviral drugs as post-exposure prophylaxis (PEP) is an established tool to prevent infection with HIV after high-risk exposure.^1^ HIV-PEP comprises a three-drug regimen administered for 28 d.^2^ In order to be maximally effective, HIV-PEP should be initiated as swiftly as possible after a potential exposure.^3^ When initiated within 72 h of exposure, HIV-PEP may reduce the risk of HIV transmission by up to 85%.^4^ However, if the time elapsed since exposure exceeds 72 h, the start of HIV-PEP is generally not recommended.^5^ The prevalence rate of people living with HIV/acquired immunodeficiency syndrome (AIDS) among the adult German population has been estimated at 0.1%.^6^ The HIV-PEP guideline currently in effect in Germany is the *Deutsch-Österreichische Leitlinie zur medikamentösen Postexpositionsprophylaxe (PEP) nach HIV-Exposition (Version 2022)* [German–Austrian Guideline for Post-Exposure Prophylaxis of HIV Infection (Version 2022)^5^]. The guideline addresses HIV transmission risk and provides recommendations for/against HIV-PEP after occupational and non-occupational potential HIV exposure.^5^

An exposure that has the potential for HIV transmission is considered a medical emergency because, as demonstrated in non-human primate models, infection with HIV is established within 24–36 h after exposure.^7,8^ Consequently, emergency departments (EDs) often serve as access points for evaluation and commencement of HIV-PEP. Accordingly, ED physicians are responsible for identifying indications but also contraindications for HIV-PEP under time pressure and need to perform a rapid, yet diligent benefit–risk analysis. On the one hand, ED physicians must consider what the potential harm to the patient is if PEP is not instituted. On the other hand, inappropriate prescribing of HIV-PEP may entail adverse drug reactions (ADRs) and cause unnecessary healthcare expenditures.^9^ According to the German–Austrian Guideline for Post-Exposure Prophylaxis of HIV Infection (Version 2022), HIV-PEP is indicated after percutaneous needlestick injuries or cut injuries that involve body fluids with potentially high HIV concentrations (e.g. blood, cerebrospinal fluid), after unprotected anal or vaginal intercourse (receptive or insertive) if the viral load of a sexual partner with known HIV infection is >1000 copies/mL or treatment status cannot be determined, after deep bleeding bite injuries by an HIV-positive person who is not or not adequately treated antiretrovirally and who has bleeding injuries in the mouth at the time of the bite (e.g. tongue bite during epileptic seizure) and after shared use of HIV-contaminated injection equipment.^5^ In addition, the guideline describes several scenarios in which HIV-PEP can be offered to patients, for instance, after unprotected anal or vaginal intercourse when there is an increased risk that the sexual partner may have an unknown or untreated HIV infection, e.g. in men who have sex with men, or in heterosexual persons if the sexual partner is from an HIV high-prevalence region (especially sub-Saharan Africa), is bisexual or is a person who injects drugs.^5^

The existing literature on HIV-PEP mainly centers on adherence to HIV-PEP regimens and subsequent seroconversion rates (i.e. treatment success/failure), rather than the appropriateness of prescriptions per se. Data from EDs about the validity of HIV-PEP prescriptions in terms of guideline concordance are limited and the rate of inappropriate HIV-PEP recommendations differs substantially from 1%^10^ to 22%.^11^

To address this ambiguity, we sought to examine the concordance of ED physicians’ clinical decisions on HIV-PEP with national guidelines in the ED of an urban tertiary care university hospital over the course of 2 y (primary objective). Additionally, we investigated post hoc the prevalence and potential impact of drug–drug interactions (DDIs) between the HIV-PEP regimen and patients’ concomitant medication on clinical decision-making in the ED (secondary objective). Enhanced knowledge about HIV-PEP prescribing characteristics in the ED may allow treatment optimisation and improvement of pharmacotherapy safety in the future.

## Materials and methods

### Study design and setting

The present investigation was conducted as a retrospective cohort study at the ED for internal medicine of Hannover Medical School, a large tertiary care university hospital with an urban catchment area in northern Germany, between 1 January 2018 and 31 December 2019. Of note, the study population was the same as in reference,^12^ but with a different research focus (HIV-PEP prescriptions vs duplicate prescriptions). The study period 2018–2019 was selected to preclude an impact of the coronavirus disease 2019 (COVID-19) pandemic on ED statistics (the first COVID-19 case in Germany was confirmed on 27 January 2020^13^).

Patients considered eligible for HIV-PEP by the treating ED physician received a starter pack of antiretroviral drugs (emtricitabine plus tenofovir disoproxil fumarate plus raltegravir) for 1–2 d. The patients were instructed to visit an outpatient infectious diseases specialist to obtain further HIV-PEP prescriptions to complete the required 28-d regimen.

We used the Strengthening the Reporting of Observational Studies in Epidemiology (STROBE) cohort checklist^14^ when planning our study and writing our manuscript (Supplementary Material).

### Eligibility criteria

Patients were eligible for enrolment in the study: 1. if they presented to the ED for internal medicine of Hannover Medical School between 1 January 2018 and 31 December 2019 after a potential exposure to HIV (sexual, occupational, accidental exposure, non-sexual assault); 2. if they were ≥18 y old; and 3. if they or their legal representative had provided written informed consent that patient-related data be used for clinical–epidemiological research. Patients were excluded: 1. if they left the ED before medical examination; or 2. if they were already taking HIV-PEP when presenting to the ED.

Of note, healthcare professionals employed at Hannover Medical School who experience a potential exposure to HIV at work (e.g. needlestick injury) present to the ED for trauma surgery (not the ED for internal medicine) and were therefore not included in this study.

### Data acquisition

The study population was identified and patient-related data (e.g. demographic characteristics, diagnoses, medications, et cetera) were provided by Hannover Medical School Information Technology via Enterprise Clinical Research Data Warehouse (ECRDW), one of the largest medical data repositories worldwide.^15^

### Outcomes

The primary outcome of our study was concordance of ED physicians’ decisions on HIV-PEP with national guidelines (for reference documents, see next paragraph). The secondary outcome was prevalence and clinical relevance of potential drug–drug interactions between the HIV-PEP regimen and patients’ concomitant medication. Potential drug–drug interactions were analysed *post hoc* by the study authors.

### Reference documents

The *Deutsch-Österreichische Leitlinien zur Postexpositionellen Prophylaxe der HIV-Infektion (update 2018)* (German–Austrian Guidelines for Post-Exposure Prophylaxis of HIV Infection (update 2018)^16^; hereafter referred to as the 2018 Guideline) served as the reference document during the study period to evaluate ED physicians’ decisions on HIV-PEP. In addition, we investigated potential clinically relevant differences between the 2018 Guideline and its successor, the *Deutsch-Österreichische Leitlinie zur medikamentösen Postexpositionsprophylaxe (PEP) nach HIV-Exposition (Version 2022)* (German–Austrian Guideline for Post-Exposure Prophylaxis of HIV Infection (Version 2022)^5^; hereafter referred to as the 2022 Guideline).

Besides editorial modifications, the main changes between the 2018 Guideline and the 2022 Guideline consisted of new recommendations on HIV-PEP after rape/sexual assault and after bite and serial cut injuries.^5^

### Drug–drug interactions

We applied the University of Liverpool HIV Drug Interactions tool^17^ post hoc to screen for and to evaluate potential DDIs between the HIV-PEP regimen and patients’ concomitant medication.

### Statistical analyses

We used descriptive statistical methods to summarise the data. Characteristics of the study population are displayed as absolute and relative frequencies for categorical variables and as medians with IQRs for quantitative variables due to non-Gaussian distribution. Statistical analyses were performed using IBM SPSS Statistics 28 (Armonk, New York, USA).

## Results

### Characteristics of the study population

**Figure 1.**
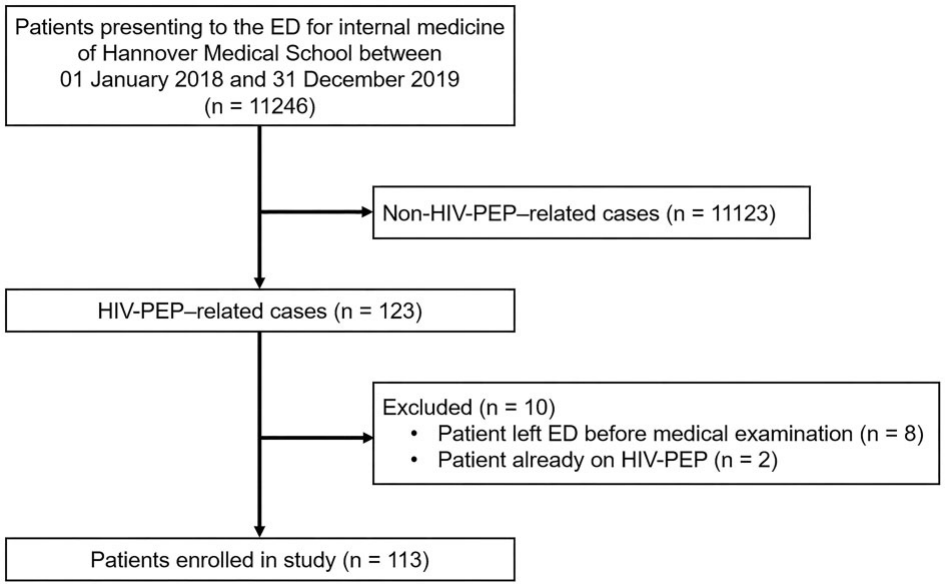
Flow of patients. Of 11 246 screened patients, 113 met the eligibility criteria and were enrolled in the study. ED: emergency department; PEP: post-exposure prophylaxis.

Of 11 246 patient cases identified via ECRDW, 123 were related to HIV-PEP (i.e. cases in which patients presented to the ED after an occupational or non-occupational potential HIV exposure and in which ED physicians had to make a decision whether HIV-PEP was indicated). Ten of these 123 cases did not meet the eligibility criteria and were excluded from further analysis, yielding a study population of n=113. The reasons for exclusion are depicted in Figure 1. The most frequent reason for exclusion was patients who left the ED before medical examination, e.g. due to waiting times.

The median age of the study population (n=113) was 30 y (IQR 25–35.5 y; range 19–62 y); 82.3% (93/113) were male (Table 1). In 85.8% (97/113), the circumstances of potential HIV exposure were characterised as sexual (anal sex, n=50; vaginal sex, n=36; ≥2 sexual modalities, n=7). Thirteen cases (11.5%) of occupational potential HIV exposure were noted in the study population [healthcare professionals (not employed at Hannover Medical School), n=8; sex workers, n=2; garbage collectors, n=1; law enforcement officials, n=1; not further specified, n=1].

**Table 1.** Characteristics of the study population (n=113)

Variables	n	%
**Gender** ^a^		
Male	93	82.3
Female	18	15.9
Transgender	2	1.8
**Circumstances of potential HIV exposure**		
Sexual^b^	97	85.8
Occupational^c^	13	11.5
Non-sexual assault	2	1.8
Accidental	1	0.9
**Mechanism or modality of potential HIV exposure**		
Anal sex	50	44.2
Vaginal sex	36	31.9
≥2 Sexual modalities^d^	7	6.2
Needlestick injury	7	6.2
Smear on intact skin or mucosa	5	4.4
Smear on wounded skin or mucosa	5	4.4
Scratch or sting injury	2	1.8
Cut injury	1	0.9
**Time elapsed between potential HIV exposure and presentation to the ED**		
<24 h	74	65.5
24–72 h	22	19.5
>72 h	2	1.8
Not determined	15	13.3
**HIV status of source person** ^e^		
Positive	17	15.0
Negative	1	0.9
Unknown	95	84.1
**Number of concomitant medications**		
None	104	92.0
One	7	6.2
Two	1	0.9
Three	–	–
Four	1	0.9

ED: emergency department.

a As documented by ED physicians.

b Including five cases of non-consensual sex/sexual assault.

c Occupational exposure was defined as a potential HIV exposure that occurred while a person was at work, e.g. a needlestick injury or mucous membrane exposure in a healthcare professional, law enforcement official, garbage collector, sex worker, et cetera.

d Combinations of anal, vaginal and/or oral sex.

e As reported by patients.

Approximately two-thirds of the study population (65.5%; 74/113) presented to the ED within 24 h after potential HIV exposure and 85.0% (96/113) presented within 72 h. The HIV status of the source person (e.g. sexual partner, assailant, et cetera) was positive in 15.0% (17/113) of cases but unknown in 84.1% (95/113). Two source persons were people who inject drugs. The majority of patients (92.0%; 104/113) did not take concomitant medication.

### Sexual exposures

Since the circumstances of potential HIV exposure in our study population were predominantly sexual, the different modalities of sexual exposure were further explored. Of note, the two cases in which sex workers were involved (designated as occupational potential HIV exposures in Table 1) were considered as sexual exposures in the subsequent analysis, leading to a total of 99 sexual exposures. Sexual exposures were evenly distributed between heterosexual intercourse and men who had sex with men [each 48.5% (48/99)]. Besides, we detected a small proportion of men who had sex with transgender women [3.0% (3/99)]. In two cases, more than two people were engaged in sexual activities. Five cases of non-consensual sex/sexual assault were noted.

Fifty-six instances of anal sex occurred in the study population (insertive anal sex, n=19; receptive anal sex, n=18; both insertive and receptive anal sex, n=2; anal sex, not further specified, n=17).

In 18 instances, male patients presented to the ED following sexual intercourse with sex workers (female or transgender women). In 1 of these 18 cases, the sex worker came from an HIV high-prevalence country [defined by the Robert Koch Institute (i.e. the German federal public health institute responsible for disease control and prevention) as countries/regions in which more than 1% of the population aged between 14 and 49 y are infected with HIV and in which heterosexual transmission is the predominant mode of transmission^18^] in sub-Saharan Africa. In seven cases, the sex workers came from HIV non-endemic regions (i.e. regions not fulfilling the definition of HIV high-prevalence countries/regions stated above). In the remaining 10 cases, the sex workers’ geographic origins were unknown.

### Safer-sex practices

Safer-sex practices were evaluated in 94 of 99 sexual exposures. In the remaining five sexual encounters, safer-sex practices were not applicable (kissing only, n=2; accidental smear of semen on scrotal bite lesion, n=1; insertion of semen-contaminated finger into sexual partner's anal canal, n=1; accidental smear of vaginal secretion on penis subsequent to condom removal after the end of sexual activity, n=1).

Condomless sex was performed in 56.4% (53/94) of sexual exposures, while condom dysfunction/failure (e.g. rupture, slippage) was noted in 36.2% (34/94). Inconsistent use of condoms (e.g. a condom was used at the onset of sexual intercourse but was later removed during sexual activity) was present in 3.2% (3/94) of sexual exposures. In 4.3% (4/94) of sexual exposures, condoms were applied correctly.

### Concordance of ED physicians’ clinical decisions on HIV-PEP with national guidelines

ED physicians recommended initiation of HIV-PEP in 71 of 113 cases (62.8%; Table 2). In 65 of these 71 cases, HIV-PEP was indicated according to national guidelines.^5,16^ Importantly, no differences were noted between the 2018 Guideline^16^ and the 2022 Guideline^5^ on the level of individual case assessments. In 42 of 113 cases (37.2%), ED physicians did not recommend HIV-PEP. In 41 of these 42 cases, HIV-PEP was not indicated according to national guidelines.^5,16^ Taken together, ED physicians’ clinical decisions on HIV-PEP were concordant with national guidelines^5,16^ in 93.8% (106/113). In seven cases (6.2%), ED physicians’ clinical decisions were discordant with national guidelines.^5,16^ In six of these seven guideline-discordant cases, HIV-PEP was recommended by ED physicians while not being indicated according to national guidelines.^5,16^ One case was detected in which HIV-PEP was not recommended by ED physicians although it was indicated according to national guidelines.^5,16^ The seven guideline-discordant cases are described in detail in Table 3.

**Table 2.** Concordance/discordance of emergency department physicians’ clinical decisions on HIV-PEP with national guidelines.^5,16^ All values are absolute frequencies

		ED physicians’ clinical decisions		
		HIV-PEP recommended	HIV-PEP not recommended	Total count
2018 Guideline^a^/2022 Guideline^b^	HIV-PEP indicated	65	1	66
	HIV-PEP not indicated	6	41	47
Total count		71	42	113

ED: emergency department; PEP: post-exposure prophylaxis.

a German–Austrian Guidelines for Post-Exposure Prophylaxis of HIV Infection (update 2018).^16^

b German–Austrian Guideline for Post-Exposure Prophylaxis of HIV Infection (Version 2022).^5^

**Table 3.** Guideline-discordant decisions on HIV-PEP by emergency department physicians. The German–Austrian Guidelines for Post-Exposure Prophylaxis of HIV Infection (update 2018)^16^ and the German–Austrian Guideline for Post-Exposure Prophylaxis of HIV Infection (Version 2022)^5^ served as reference

Type of exposure	HIV-PEP prescribed while not indicated	HIV-PEP not prescribed while indicated
Occupational	Healthcare professional: injury from a dental drill contaminated with saliva of a person with unknown HIV status	–
	Law enforcement official: injury incurred from an assault with a toothpick contaminated with saliva of a presumably HIV-positive person	
	Sex worker: heterosexual anal sex with a dysfunctional condom	
Sexual	Unprotected heterosexual anal sex with a sex worker from an HIV non-endemic region	Unprotected heterosexual anal sex with a sex worker from an HIV high-prevalence country (sub-Saharan Africa)
	Unprotected heterosexual anal and vaginal sex without risk factors for HIV	
	Unprotected heterosexual sex with a person with unknown HIV status	

PEP: post-exposure prophylaxis.

### Drug–drug interactions

Nine of 113 patients (8.0%) took concomitant medication, predominantly antihypertensive agents. No clinically relevant DDIs between the HIV-PEP regimen (i.e. emtricitabine plus tenofovir disoproxil fumarate plus raltegravir) and patients’ concomitant medication were detected.

## Discussion

The present study investigated the concordance of ED physicians’ clinical decisions on HIV-PEP with national guidelines^5,16^ in the setting of the ED for internal medicine of a large German university hospital. Approximately 1% of patient presentations to the ED were related to a potential HIV exposure, demonstrating the importance of knowledge and training of ED physicians on HIV-PEP. A review of the ED's role in HIV prevention and treatment stated that PEP is an ‘important and well-accepted part of emergency physicians’ practice’.^1^

For many people seeking help after a potential HIV exposure, the ED is the primary access point to medical care due to its 24-h availability. Studies show that people seek treatment at EDs after potential HIV exposures with increasing frequency.^1^ In our study, 85% of patients presented within 72 h of potential HIV exposure, which represents the time frame according to national guidelines within which initiation of HIV-PEP is pathophysiologically reasonable.^5,16^ Commencement of HIV-PEP is time-sensitive and is most effective if the first dose is administered as quickly as possible after exposure, ideally within the first 2 h.^19^ To the best of our knowledge, our study is the first to evaluate the concordance of ED physicians’ clinical decisions with the German–Austrian HIV-PEP guidelines.^5,16^

ED physicians recommended initiation of HIV-PEP in nearly two-thirds of HIV-PEP-related cases in our study, which is comparable to other reports.^10,11,20,21^ ED physicians’ clinical decisions were concordant with national guidelines^5,16^ in 93.8%. Modifications and updates between the 2018 Guideline^16^ and the 2022 Guideline^5^ had no influence on the concordance rate of ED physicians’ clinical decisions on HIV-PEP, suggesting that the changes implemented between 2018 and 2022 were of minor clinical relevance in ED routine. The prototypical patient who was prescribed HIV-PEP in our study was a man who had unprotected anal sex with another man. The prototypical patient who was not prescribed HIV-PEP was a man who had unprotected vaginal sex with a woman. The majority of potential HIV exposures in our study were to sources of unknown HIV status (84.1%), which is similar to an investigation by Merchant and co-workers.^22^ In contrast to our study, however, the appropriateness of HIV-PEP prescriptions was not assessed in the Merchant et al. report due to missing information in ED documentation.^22^

In another study by Merchant et al., in which 3622 patient visits for blood or body fluid exposures at 12 EDs in Rhode Island, USA, from 1995 to 2001 were reviewed, 48.0% of exposures were sexual^23^ vs 85.8% in our study. The majority of patients who were sexually exposed in the Merchant et al. study were female (89.9%) and 95.8% of sexual exposures were due to sexual assault,^23^ which stands in stark contrast to our study, in which only 8 of 97 patients who were sexually exposed were female and 92 of 97 sexual exposures were consensual. HIV-PEP was offered to only 21.0% of patients in the Merchant et al. study^23^ vs 62.8% in our study. Demographic differences, but also changes in HIV-PEP guidelines over time, may account for these discrepancies.

Malinverni and colleagues observed a low rate of inappropriate HIV-PEP prescriptions (1.2%) according to BREACH (Belgium Research on AIDS and HIV Consortium) guidelines on non-occupational PEP in a retrospective study conducted at an ED in Brussels, Belgium.^10^ Of note, ED physicians had the possibility to consult an infectious diseases specialist around the clock in cases of doubt, which might explain the low rate of inadequate HIV-PEP prescriptions. The authors did not comment on the health-economic costs of their standard operating procedure, which might limit transferability to other less affluent healthcare systems, especially in low-income countries.

Several studies from the United Kingdom,^24–26^ which shall be discussed in the following, assessed the concordance of decisions on HIV-PEP based on BASHH (British Association for Sexual Health and HIV) guidelines. Of note, pre-2021 BASHH guidelines (as applied in those studies^24–26^) were only applicable to PEP following sexual exposure. Although the majority of potential HIV exposures in our study were sexual (85.8%), we also included other types of exposure (e.g. occupational). Therefore, the subsequent comparisons of guideline concordance/discordance of studies from the United Kingdom that applied pre-2021 BASHH guidelines^24–26^ with our results must be interpreted with circumspection. In a retrospective monocentric study conducted in Belfast,^24^ guideline concordance was similar to our study (93%^24^ vs 93.8%). The proportion of patients presenting within 72 h of potential HIV exposure, however, was slightly higher (92%^24^ vs 85%). The rate of guideline-discordant decisions on HIV-PEP was low (1.9%) in a retrospective monocentric study from London.^25^ While the proportion of male patients (97.7%) and the proportion of patients who presented within 72 h of potential HIV exposure (99.8%)^25^ were notably higher than in our study (82.3% and 85.0%, respectively), the rates of condomless sex (55.0%) and condom dysfunction (31.8%)^25^ were comparable to our analysis (56.4% and 36.2%, respectively). In a case-note review of 101 patients at a genitourinary clinic in the West Midlands,^26^ the rate of sexual assault (30.7%) was considerably higher than in our study. While the proportion of male patients was substantially lower than in our investigation (48.5%^26^ vs 82.3%), the proportion of patients who presented within 72 h of potential HIV exposure was similar (84.2%^26^ vs 85.0%). Also, 82.2% of HIV-PEP prescriptions were guideline-concordant,^26^ which was markedly lower than in our study (93.8%).

Marzel and colleagues conducted a retrospective cross-sectional study on the appropriateness of HIV-PEP prescriptions in Zurich, Switzerland.^11^ The sample size was larger than in our investigation (n=975^11^ vs n=113). However, our study cohort was more diverse since we did not exclude occupational exposures or cases of sexual assault/non-consensual sexual exposures. In the Marzel et al. study, HIV-PEP was prescribed in 61%, and 74% of decisions were guideline-concordant (according to internal guidelines of the University Hospital Zurich)^11^; both proportions were lower than in our analysis (62.8% and 93.8%, respectively).

Scholten et al. evaluated decisions on HIV-PEP in an infectious diseases clinic (IDC) in Cologne, Germany.^27^ Importantly, patients who visited the ED and who were not subsequently referred to the IDC were explicitly excluded from the analysis of guideline concordance of HIV-PEP prescriptions in the Scholten et al. study.^27^ Meaningful comparisons with our report can therefore not be drawn.

Of particular interest, no clinically relevant DDIs between the HIV-PEP regimen and patients’ concomitant medication were detected in our study. The HIV-PEP regimen used at our hospital (emtricitabine plus tenofovir disoproxil fumarate plus raltegravir) does not include ritonavir, which has a much higher DDI potential and which was cited as a major contributor to HIV-PEP-related ADRs in older reports.^28^ Particularly noteworthy in this regard is the case of a 32-year-old woman in whom a drug interaction between dihydroergotamine and a ritonavir-based HIV-PEP regimen elicited severe ischemia of the left foot (ergotism),^29^ which impressively demonstrates the necessity for diligent drug interaction checks prior to initiation of HIV-PEP. Modern ritonavir-free HIV-PEP regimens are less prone to DDIs and should generally be preferred. The integrase inhibitor raltegravir is commonly prescribed as one component of the three-drug HIV-PEP regimen because of its good tolerability and low risk of DDIs.^2^ Nonetheless, the potential of raltegravir for hypersensitivity and muscle toxicity must be observed.^30^

In four of the seven guideline-discordant decisions in our study, the HIV transmission risk of heterosexual sex (vaginal and/or anal) was overestimated by the treating ED physician and HIV-PEP was prescribed while not indicated according to national guidelines.^5,16^ In two cases, the HIV transmission risk of saliva was overestimated. Saliva is not considered a body fluid with high virus concentration^5,16^; besides, saliva has been demonstrated to lyse HIV in vitro because of its hypotonicity, and certain salivary proteins have the capacity to inactivate HIV.^31^ Consequently, HIV-PEP is generally not indicated following exposure to saliva.^5,16^ In one case, the geographic origin of the source person (an HIV high-prevalence country in sub-Saharan Africa) was not taken into account during risk assessment and HIV-PEP was withheld although it was indicated.^5,16^ Taken together, our analysis of guideline-discordant decisions suggests that, in general, ED physicians appear to be rather cautious and tend to prescribe HIV-PEP in cases of doubt or ambiguity, even if not supported by guidelines. Limited time for a comprehensive risk assessment in the ED, fear of medicolegal consequences if HIV-PEP is withheld or a demanding attitude of some patients may play a critical role in this regard.

Our study population comprised a diverse representation of major HIV-PEP categories such as occupational exposures (e.g. in healthcare professionals, law enforcement officials, sex workers), men who have sex with men and non-consensual sex/sexual assault, which can be considered a strength of our report. The retrospective character and monocentric design, however, represent limitations. Our study was conducted at a single urban tertiary care university hospital, limiting its transferability to other settings. Our patient mix, patient volume, geographical location, catchment area and internal hospital policies are unique to our setting and not generalisable to other environments. Information about patients’ baseline HIV status, presence of other sexually transmitted diseases, tolerability and completion rates of HIV-PEP, or information about which patients might be appropriate candidates for pre-exposure prophylaxis (PrEP) was not available within the constraints of the present study. It must be stressed that no cases of PrEP use were registered in our study population, which might be explained by the fact that PrEP was introduced in Germany for people (with statutory health insurance) who are at substantial risk of HIV infection just at the end of our study period, in September 2019. In a future investigation, it should be determined whether patients on PrEP presenting to the ED are significant in number, and whether PEP is prescribed appropriately in this context.

Perhaps the most significant limitation of our study is a lack of information about the number of instances in which ED physicians’ clinical decisions on HIV-PEP yielded the desired clinical outcome, namely absence of seroconversion. This assessment was beyond the scope of our analysis. Nevertheless, our study opens avenues for further research. We propose that future investigations should enroll a larger number of HIV-PEP-related cases, preferably within a multicentric approach, and that these cases should be followed up prospectively to allow greater completeness of data, particularly with regard to clinical outcomes.

## Conclusions

In this analysis of 113 ED visits following potential HIV exposure, physicians prescribed HIV-PEP in concordance with national guidelines in 93.8% of cases, demonstrating that adherence to HIV-PEP guidelines is feasible even in the hectic environment of an ED. A diligent screening for potential DDIs between the HIV-PEP regimen and patients’ concomitant medication is indispensable, although we did not detect clinically relevant DDIs in our young and predominantly unmedicated study population. ED physicians may benefit from specialised training on HIV transmission risk assessment and indications/contraindications for HIV-PEP.

## Supplementary Material

ihad076_Supplemental_File

## Data Availability

The data underlying this article will be shared on reasonable request to the corresponding author.
